# Adult Wilms tumor with inferior vena cava thrombus and distal deep vein thrombosis – a case report and literature review

**DOI:** 10.1186/s12957-018-1343-4

**Published:** 2018-02-23

**Authors:** Krzysztof Ratajczyk, Adrian Czekaj, Joanna Rogala, Pawel Kowal

**Affiliations:** 1 Department of Urology, Regional Specialist Hospital, Wroclaw, Poland; 2 Department of Pathology, Regional Specialist Hospital, Wroclaw, Poland

**Keywords:** Adult Wilms tumor, Vena cava inferior, Ligation, Venous thrombosis

## Abstract

**Background:**

Adult Wilms tumor (WT, nephroblastoma) is a rare, but well-described renal neoplasm. Although inferior vena cava tumor thrombosis is present in up to 10% of Wilms tumors in childhood, only few cases of this clinical manifestation in adults have been reported. To the best of our knowledge, this is the first case of adult WT infiltrating into inferior vena cava (IVC) with concomitant distal deep vein thrombosis.

**Case presentation:**

A 28-year-old male patient with gross hematuria and right flank pain was diagnosed with right kidney tumor penetrating to IVC. Preoperatively, acute distal thrombosis in inferior vena cava and lower extremities veins occurred. Right radical nephrectomy with tumor thrombectomy via cavotomy was performed. In order to prevent pulmonary embolism, IVC was ligated below left renal vein level. Histopathological examination revealed a triphasic nephroblastoma without anaplastic features. Postoperatively, patient was diagnosed with metastatic liver disease, which was treated with two lines of chemotherapy followed by radiotherapy with achievement of complete response.

**Conclusions:**

Adult WT occurs usually in young patients, under 40 years of age. Neoadjuvant chemotherapy proved to be effective in children, resulting with tumor shrinkage and venous tumor thrombus regression. Therefore, percutaneous biopsy should be always considered in young patients presenting with renal tumor invading venous system. IVC ligation is a safe treatment option in the event of complete inferior vena cava occlusion due to distal thrombosis concomitant to tumor thrombus, provided collateral venous pathways are well-developed.

## Background

Wilms tumor (WT, nephroblastoma) is the most common renal neoplasm in children, but can rarely occur in adults, with an incidence of 0.2 per million (70 newly diagnosed cases yearly in Europe) [1]. In the English literature, more than 300 cases of adult Wilms tumor were reported, with only few describing inferior vena cava (IVC) involvement [2, 3].

In this report, we discuss the first case of an adult patient with Wilms tumor infiltrating into inferior vena cava with concomitant distal deep vein thrombosis.

## Case report

**Fig. 1:**
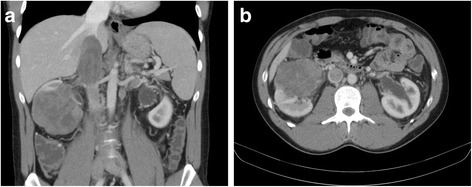
Abdominal CT scan at the time of diagnosis. Right kidney tumor with inferior vena cava tumor thrombus extending to the hepatic veins’ level. **a** Coronal plane. **b** Transverse plane

A 28-year-old male was admitted to the emergency department with right flank pain and gross hematuria. Ultrasound revealed 8 cm right renal mass. Ambulatory computed tomography (CT) scan showed a 7.8 × 6.8 × 8.5 cm, contrast-enhancing tumor in the right kidney with IVC thrombus extending to the level of hepatic veins. (Fig. 1) Elective surgery was scheduled. However, the patient attended ER ward 5 days before scheduled admission date due to lower limbs edema and pain. Doppler ultrasound revealed massive acute deep vein thrombosis in lower limbs and vena cava below renal veins level. Anticoagulation therapy with low-molecular-weight heparin (LMWH) was administered. Patient was scheduled for urgent surgical treatment. Right radical nephrectomy with complete vena cava tumor thrombus resection using liver transplantation techniques (i.e., complete liver mobilization, suprahepatic IVC clamping, Pringle maneuver and wide cavotomy) was performed. Due to failure of the distal embolectomy, IVC was ligated below renal veins in order to prevent pulmonary embolism. During surgery, no gross invasion of the adjacent structures or regional lymphadenopathy was noted. The operative time was 300 min and estimated blood loss was 2000 ml. Patient recovered well and was discharged at sixth day postoperatively with permanent anticoagulation therapy with weight-adjusted curative doses of LMWH.

**Fig. 2:**
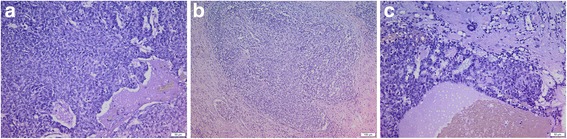
Triphasic pattern of Wilms tumor. **a** Predominant blastemal cells (H/E, 20×). **b** Stromal component (H/E, 10×). **c** Minor epithelioid component (H/E, 20×)

On gross examination the tumor was 9 cm large, white, solid/cystic, with soft cut-surface, unencapsulated, but infiltrating adipose tissue. Histopathological examination revealed a triphasic nephroblastoma composed predominantly of blastema and non-specific spindle cell stroma with minor epithelioid component. (Fig. 2) No features of anaplasia were found. Immunohistochemistry showed positivity for WT1, CD99, CD57, INI1, CKAE1/AE3, VIM, EMA, SMA, Bcl-2, and PAX-8. HMWCK, CK20, CK7, AMACR, SOX10, MDM2, desmin, MYOD1, caldesmon, myogenin, CD34, and S100 were negative. The tumor was classified as favorable risk group with pathologic stage III according to the Children’s Oncology Group (COG) staging system (Table 1) [4].

**Table 1:** Children Oncology Group (COG) staging system for Wilms tumor

Stage	Criteria
I	Tumor limited to kidney and completely resected with negative surgical margins. Renal capsule not involved. No tumor biopsy or rupture prior to removal. Renal vessels and hilar fat not involved.
II	Tumor extension beyond the kidney—to perinephric fat or to the vessels. Tumor resected completely with negative surgical margins.
III	Residual tumor after surgery:
	-Abdominopelvic lymph nodes involvement
	-Presence of tumor cells at the resection margins
	-Tumor biopsy or rupture before or during surgery
	-Tumor implants on the peritoneal surface
	-Irresectable tumor due to local vital organs involvement
IV	Hematogenous metastases or lymph node metastases outside the abdominopelvic region.
V	Bilateral renal involvement by tumor at the diagnosis.

**Fig. 3:**
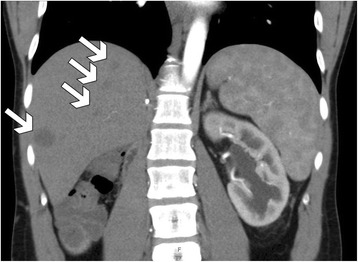
Abdominal CT scan at 3 months postoperatively-multiple liver metastases (*white arrows*)

At follow-up visit after 3 months, the patient presented with mild lower limbs edema. On abdominal CT scan, more than 20 liver metastases were identified (Fig. 3). According to the strategy proved to be effective in children, patient was scheduled to receive adjuvant chemotherapy with two courses of actinomycin D, vincristine, and doxorubicin. Due to insufficient response, second-line chemotherapy with doxorubicin, cyclofosfamide, etoposide, and carboplatin followed by liver irradiation was administered. CT scan performed 18 months postoperatively has shown complete response with no residual tumor lesions. At last follow-up, the patient presented with mild right lower limb edema, dilated abdominal wall veins and abdominal hernia in the midline of postoperative scar after Mercedes-Benz incision.

## Discussion

Wilms tumor is the most common renal neoplasm in childhood, but is responsible for less than 1% of renal tumors in adulthood [5]. Due to its rarity in the adult population, it is not taken into consideration preoperatively and as such would not be given a chance of preoperative chemotherapy. The difficulty in diagnosing such tumors in the adult populations with subsequent postoperative chemotherapy delay does adversely influence the prognosis, which is worse in adults comparing to children [6].

Venous involvement of WT in the childhood is frequent and occurs in up to 45% of cases. In IVC, tumor thrombosis is present in 4–10% of patients, with 1–3% of right atrium involvement [7–10]. Although these figures well correspond with IVC tumor thrombosis rate in adult renal cell carcinoma (RCC), the incidence of venous involvement in adult WT is unknown. So far there are only few case reports addressing this area in the literature [2, 3, 11].

In pediatric patients, there are two main approaches to diagnosis and treatment of WT. The Children’s Oncology Group (COG) protocols, utilized in North America, stresses the importance of primary surgical treatment with the emphasis on precise pathological evaluation before chemotherapy and radiotherapy [5]. In Europe, WT is treated according to the International Society of Pediatric Oncology (SIOP) protocols, which propose neoadjuvant chemotherapy for 4–6 weeks, in most cases, without pretreatment tumor biopsy. This approach has shown to reduce tumor size and intraoperative tumor rupture rates, thus, decreasing difficulty of the surgery and local recurrence rates. However, lack of histopathological diagnosis holds a risk of unnecessary chemotherapy administration in patients with benign or non-WT tumors [12].

Both aforementioned groups (COG and SIOP) agreed that patients with WT infiltrating IVC and right atrium of the heart would benefit from preoperative chemotherapy. Majority of tumor thrombi respond to neoadjuvant treatment [8, 10]. Tumor thrombus shrinkage in many cases decreases difficulty of the surgery, thus, lowering the risk of perioperative complications. Shamberger et al. achieved a tumor thrombus regression in 39 of 49 patients after 8 weeks of neoadjuvant chemotherapy. In 7 of 12 patients, thrombus regressed from atrial location, thus, eliminating the need for cardiopulmonary bypass (CPB) [8]. Hadley et al. described measurable thrombus regression in 18 of 40 patients with 3 patients avoiding the need for CPB [10]. Another study group presented complete response in 2 patients and partial response in 6 of 11 patients submitted to preoperative chemotherapy [13]. Although these results seem satisfactory, there is a group of patients that could be harmed from delayed surgery. Complications and deaths due to pulmonary embolism, tumor progression, and treatment toxicity during preoperative chemotherapy phase have been described [8, 14]. Choice of treatment regimen should be considered individually. Immediate surgical treatment is advised in the event of unstable tumor thrombus, a patient with acute cardiac failure or Budd–Chiari syndrome at presentation [7, 13].

Percutaneous needle core biopsy under ultrasound or CT guidance is a safe and accurate procedure. Post-biopsy complication rate is low (1%), with incidental cases of tumor seeding along biopsy tract [15].

According to the data from EUROCARE study, median age at the presentation of adult Wilms tumor is 34 years [1]. Eggener et al. reported the incidence of benign histology on final pathological report in more than 20% of young adults, aged 17 to 45, with contrast-enhancing renal masses treated with partial or radical nephrectomy [16]. Other rare malignant renal tumors, including primitive neuroectodermal tumor (PNET) and synovial sarcoma should be also taken into consideration in the differential diagnosis [17, 18]. Therefore, preoperative core biopsy should be considered in young adults (18–40 years) to establish proper pathological diagnosis. This procedure would be beneficial in eliminating the need for surgery in the presence of benign histology or giving opportunity to apply multimodal treatment in case of WT or other non-RCC malignant tumors. This approach would be especially advised in tumors extending to IVC and right atrium of the heart. Histopathological diagnosis of WT from biopsy would allow introduction of preoperative chemotherapy, which is proved to be efficient in achieving tumor shrinkage, thrombus retraction, and minimizing intraoperative tumor spillage. Altogether, it would make the surgery easier for the surgeon and safer for the patient.

In pediatric patients, the introduction of adjuvant chemotherapy is recommended within 1–2 weeks post nephrectomy [6]. In adult series of 17 patients, median time to postoperative chemotherapy administration was 59 days. The patients who had it introduced within the first 30 days after surgery presented with significantly better 5-year overall survival (80%) than the group of patients with delayed therapy induction (28.6%) [19]. Therefore, the delay of adjuvant treatment introduction may be partially responsible for worse treatment outcome in adult WT patients. In our case, due to prolonged histopathological consultations, patient received first dose of chemotherapy 90 days after surgical treatment. On the basis of this experience, we recommend needle biopsy before definitive treatment in every case of young patient with the renal mass. This is of the greatest importance in subset of patients with IVC tumor thrombosis. In this group, early definite histopathological diagnosis may facilitate the surgery, while its delay can result in disease progression, serious comorbidities, or even death.

IVC thrombosis is uncommon and usually associated with malignant disease, congenital IVC malformations, or untreated IVC filter [20, 21]. Tumor thrombus extending from renal neoplasm to IVC can be accompanied by bland (i.e., consisting clotted blood) thrombus. The latter reaches in most cases below the iliac bifurcation and may be followed by symptomatic pulmonary embolism in up to 27% of cases [18, 19]. The incidence of the concomitant bland thrombus was found to be higher in more advanced levels of tumor thrombus [22].

Symptoms of acute IVC thrombosis may vary with severity, from mild (i.e., lower limbs edema, lower back pain) to life-threatening (i.e., cardiovascular failure due to impaired venous return or pulmonary embolism). If intrahepatic IVC or renal veins are involved, patient may present symptoms of renal congestion and Budd–Chiari syndrome (i.e., hepatomegaly, ascites, and jaundice) [23–25]. Patients with chronic IVC obstruction develop venous collaterals diagnosed either radiographically (e.g., dilated lumbar-hemiazygos veins), or clinically (e.g., dilated abdominal wall veins) [22, 26]. The presence of collateral venous pathways, deep and superficial, is responsible for the lack of severe IVC obstruction symptoms. The deep pathway, comprising of ascending lumbar veins, anastomosing with the azygos and hemiazygos veins, plays a crucial role in venous decompression, irrespective of the level of obstruction. This pathway consists of vertebral venous plexus, left gonadal vein (provided that left renal vein is patent), periureteric plexus, and portal vein system (i.e., through hemorrhoidal plexus, inferior mesenteric vein and splenic vein or through recanalized paraumbilical veins, ductus and hepatic vein). In the superficial pathway, blood flows through the inferior epigastric veins (via superior epigastric, internal mammary, and subclavian veins) and the circumflex iliac veins (via superficial epigastric, lateral thoracic, and axillary veins) to ultimately reach superior vena cava [27, 28].

Several factors, such as degree, level, and duration of venous obstruction, presence of collateral vessels should be evaluated before the treatment to determine the feasibility and extent of the surgery. Resection of IVC is not required as long as tumor and bland thrombi are completely removed [22]. However, when IVC resection is necessary, its internal diameter can be decreased by 50% without significant blood flow impairment [26, 29]. Small cavotomies can be closed primarily with running 4–0 suture as well as with the application of autologous or prosthetic patch [26]. Several surgical interventions preventing propagation of bland thrombus after tumor thrombus excision were described (i.e., IVC ligation, segmental IVC resection and reconstruction, IVC filter placement). The interruption of the IVC can be achieved by either stapling, ligation, or oversewing [22, 30]. Ayyathurai et al. [22] suggested surgical interruption in the following situations:

- Tumors involving more than half of IVC circumference,
- Complete chronic IVC obstruction in the absence of venostasis clinical symptoms,
- High risk of postoperative pulmonary embolism due to residual irresectable bland thrombus, and
- Successful thrombectomy complicated by vascular intima layer damage causing increased risk of new clot development.

Postoperative morbidity after IVC interruption is minimal, provided the presence of chronic obstruction [22]. However, surgical IVC ligation with undeveloped collateral pathways may lead to symptoms as in acute IVC thrombosis. One of the keys to minimizing morbidity is to avoid collateral vessels ligation [31]. Left renal vein can be transected proximally to anastomosing with its posterior aspect lumbar vein, thus, sustaining the connection to the hemiazygos-azygos system [25, 32]. Left renal vein pressure measurement was proposed to determine efficiency of collateral venous pathways. Pressure in renal vein below 40 mmHg means that the blood flow through the venous collaterals is sufficient to avoid renal congestion [32].

Patients presenting with clinical symptoms of IVC obstruction, with insufficient venous collaterals or intraoperative dissection of preexisting collaterals, should be considered for IVC reconstruction with application of polytetrafluoroethylene (PTFE) graft, bovine pericardium, or autologous vein graft (e.g., paneled vein graft, superficial femoral vein, cryopreserved vein graft) [29, 30].

In our patient, due to complete IVC obstruction by tumor thrombus accompanied by bland thrombus reaching below the IVC bifurcation, and in the absence of clinically significant venostasis symptoms, we decided to perform complete IVC ligation below left renal vein level, with no postoperative complication. However, according to Ayyathurai et al. [22], patients after surgical IVC interruption are at higher risk of intraoperative and postoperative complications, not influenced by the presence of bland thrombus itself. In his series, among 129 patients with renal malignancy and tumor thrombus, 15 patients (12%) had concomitant bland thrombus. The IVC ligation was performed in 8 patients (53%) with bland thrombus, compared to 11 patients (10%, *p* < 0.001) without distal thrombosis [22].

In the series of Blute et al., 40 of 160 patients (25%) underwent IVC interruption. In this group, 23 patients (14.4%) had IVC ligated, 4 (2.5%) had IVC filter placed for co-existing bland thrombus and 13 patients (8.1%) had IVC segmentally resected due to venous wall infiltration [31].

The main indicators for IVC interruption are the presence of bland thrombus and the degree of venous occlusion [22, 31].

## Conclusions

Adult Wilms tumors are rare but occur usually in young adults. Neoadjuvant chemotherapy proved to be effective in children, resulting in tumor shrinkage and venous tumor thrombus regression. As such, image-guided percutaneous core biopsy is strongly advised in all young adults, under 40 years of age, presenting with renal tumor and venous system invasion.

Inferior vena cava ligation is a safe treatment option in case of complete and chronic vena cava inferior occlusion due to complete distal thrombosis concomitant to tumor thrombus, provided that collateral pathways are well developed. Primary IVC reconstruction should remain the surgical strategy of first choice in patients presenting with clinical symptoms of IVC obstruction, with insufficient venous collaterals or intraoperative dissection of preexisting collaterals.

## Abbreviations

COG: Children’s oncology group
CPB: Cardiopulmonary bypass
CT: Computed tomography
IVC: Inferior vena cava
LMWH: Low-molecular-weight heparin
PNET: Primitive neuroectodermal tumor
PTFE: Polytetrafluoroethylene
RCC: Renal cell carcinoma
SIOP: International society of pediatric oncology
WT: Wilms tumor, nephroblastoma
